# Supplementary material for: Prevalence, patterns and predictors of metabolic abnormalities in Nigerian hypertensives with hypertriglyceridemic waist phenotype: A cross sectional study
Source: PLOS Glob Public Health. 2022 Dec 16;2(12):e0001203. doi: 10.1371/journal.pgph.0001203 (PMC10021371; doi:10.1371/journal.pgph.0001203)
Supplement: Attachment — Submitted filename: Response to Reviewers (1).docx [file pgph.0001203.s003.docx]

**Response to Reviewers**

Journal Requirements:

1. In the online submission form, you indicated that "Data is available on request". All PLOS journals now require all data underlying the findings described in their manuscript to be freely available to other researchers, either 1. In a public repository, 2. Within the manuscript itself, or 3. Uploaded as supplementary information.

In line with the policy of PLOS we have declared that our data is freely available and will be uploaded as supplementary file.

2. "Please provide separate figure files in .tif or .eps format.

Figures were reviewed with PACE digital diagnostic tool and uploaded as separate files as tiff

3. Please review your reference list to ensure that it is complete and correct. If you have cited papers that have been retracted, please include the rationale for doing so in the manuscript text, or remove these references and replace them with relevant current references. Any changes to the reference list should be mentioned in the rebuttal letter that accompanies your revised manuscript. If you need to cite a retracted article, indicate the article’s retracted status in the References list and also include a citation and full reference for the retraction notice.

The references have been reviewed and amendments made in the reference list.

Reviewer’s comment #5

1. The Odds Ratio Table (Table 4) has been reviewed and all concerns and inconsistencies have been addressed and corrections made in the table 4 and reflected in the text.
2. Redundant statements in the ‘’Discussion’’ have been either edited or removed
3. ANOVA was used in Table 2 and has been added as a legend
